# Laparoscopic repair of Morgagni hernia in children

**DOI:** 10.1016/j.amsu.2020.05.012

**Published:** 2020-05-30

**Authors:** Ali İhsan Anadolulu, Gonca Gerçel, Osman Hakan Kocaman

**Affiliations:** Mehmet Akif İnan Training and Research Hospital Clinic of Pediatric Surgery, Şanlıurfa, Turkey; Şanlıurfa Training and Research Hospital Clinic of Pediatric Surgery, Şanlıurfa, Turkey; Harran University, Clinic of Pediatric Surgery, Şanlıurfa, Turkey

**Keywords:** Morgagni hernia, Children, Minimally invasive, Laparoscopic

## Abstract

**Aim:**

We aimed to present our laparoscopic treatment experience in Morgagni hernia repair.

**Methods:**

The patients who underwent laparoscopic surgery with diagnosis of Morgagni hernia between 2016 and 2019 were evaluated retrospectively.

**Results:**

Their mean age at diagnosis was 4,1 ± 2,6 years (1 year-13 years). All patients were male. The presenting complaints were respiratory tract infection in 3 patients and vomiting in 3. Two patients were diagnosed incidentally. Associated Down's Syndrome was detected in 3 (38%) cases. The defect was left-sided in 7 (87.5%) patients and bilateral in 1 (12,5%). Omentum was herniated in 2 patients, colon and omentum were in 6 and colon, omentum and stomach were in one. All patients underwent primary repair extracorporeally by removing sutures from single incision, without removal of the hernia sac. There were no complications or recurrence in the mean 19,2 ± 15,8 months (6–42 months) follow-up period.

**Conclusions:**

Minimal invasive repair of Morgagni hernia is efficient and safe. It should be the first choice because of fast recovery and better cosmetic results. In this series, it was seen that leaving the hernia sac had no effect on early and late complications. Leaving the hernia sac may prevent potential complications due to unnecessary dissection.

## Highlights

• The treatment of MH is surgical repair either conventionally by open abdominal or thoracic approaches, or more recently by minimal invasive surgery.
• Minimal invasive laparoscopic-assisted repair of MH using sutures including the full thickness of anterior abdominal wall and extracorporeal knots proved to be effective, safe, and reliable in children.
• It should be the first choice because of fast recovery and better cosmetic results.
• Leaving the hernia sac had no effect on early and late complications.
• Leaving the hernia sac may prevent potential complications due to unnecessary dissection.

## 1 Introduction

The foramen of Morgagni is a retrosternal space that develops when the fibrotendinous portion of the pars sternalis does not fuse with the fibrotendinous tissue arising from the costochondral arches [1]. A patent foramen of Morgagni offers a path through which the abdominal viscera can herniate into thoracic cavity. It was first described by Giovani Morgagni in 1769 and has unique features in terms of clinical presentation and associated anomalies [2].

Morgagni hernia (MH) is extremely rare, occurring approximately in 1 out of 5000 live births, and accounts for less than 5% of all congenital diaphragmatic defects [3,4]. Patients may present with an incidental diagnosis or nonspecific respiratory symptoms such as frequent lung infection, dyspnea or ileus and abdominal pain [[5], [6], [7], [8]]. MH may also be associated with heart defects and Down syndrome [9]. Clinical experience with this entity is limited owing to its rare occurrence. The treatment of MH is surgical repair either conventionally by open abdominal or thoracic approaches, or more recently by minimal invasive surgery [10,11]. With this study we aimed to present our experience with the laparoscopic repair of MH.

## 2 Material and methods

**Fig. 1:**
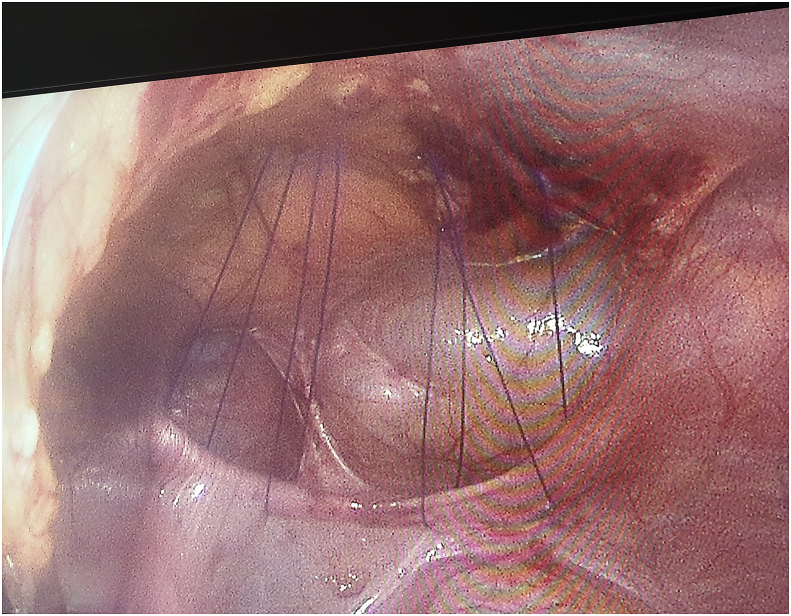
Operative view of the defect and stitches including the sac.

The medical records of the children operated on from 2016 to June 2019 with the diagnosis of MH were reviewed retrospectively. Patients were included when submitted to laparoscopic-assisted surgical approach using three ports and when sutures were performed with, separated, percutaneous, ‘‘U’’ shaped, stitches, through the full thickness of the anterior abdominal wall (Fig. 1) and the knots were tied in the subcutaneous tissue by a single incision. Surgeries were performed by 3 pediatric surgeons who experienced between 1 and 5 years. The following information was obtained: age at diagnosis, sex, presenting symptoms, method of diagnosis, associated anomalies, site of hernia, operative repair and outcome.

All procedures performed were in accordance with the ethical standards of the responsible committee on human experimentation (institutional and national) and with the Helsinki Declaration of 1964 and later versions. This study has been reported in line with the PROCESS 2018 criteria [12]. Written informed consent was obtained from the patients for the surgery and information to be included in our manuscript.

## 3 Results

**Fig. 2:**
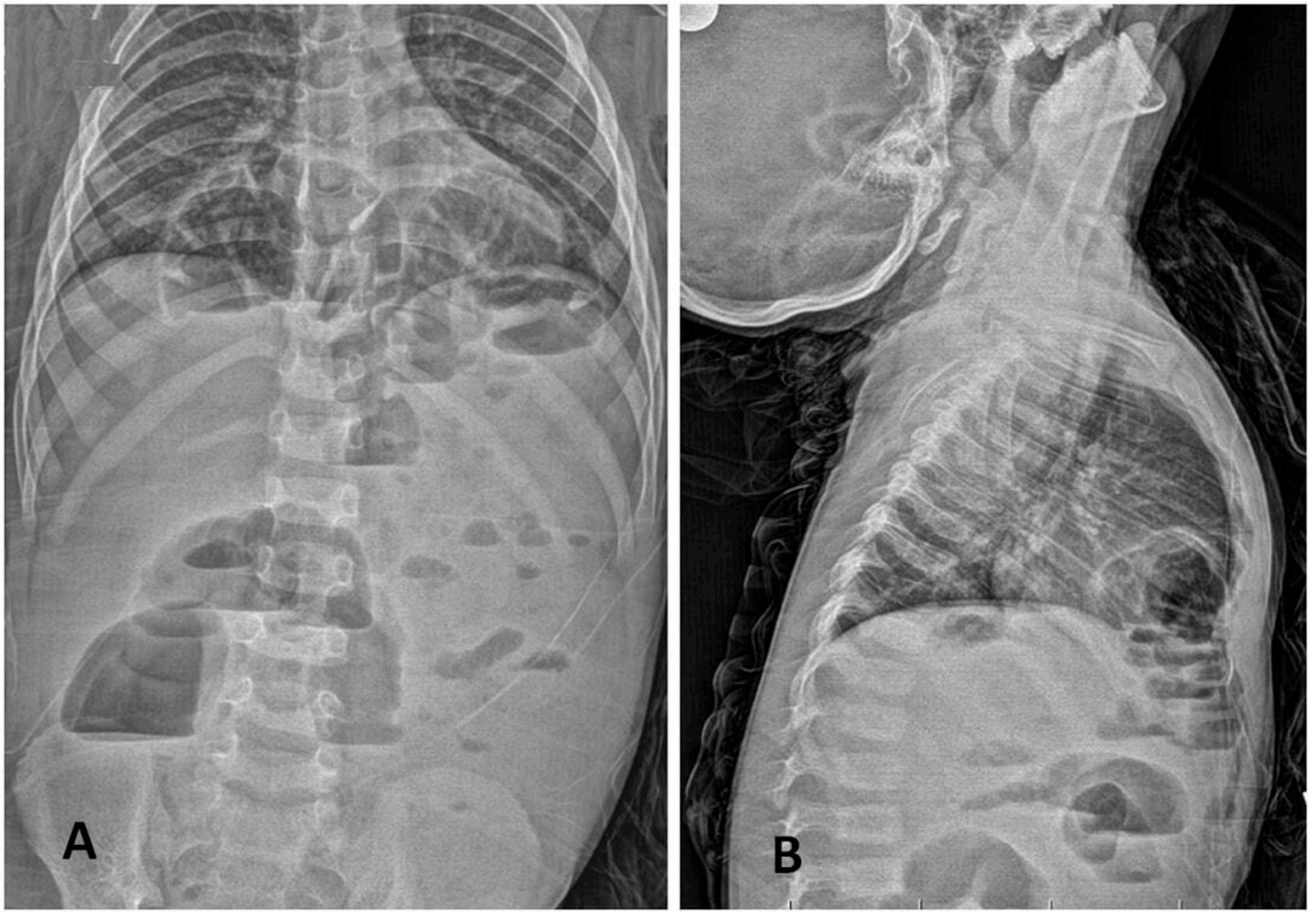
Anteroposterior (A) and lateral (B) chest x-ray showing anterior herniation of bowel loops into the chest and abdominal air fluid levels.

During the study period, 8 male children with MH were operated. Their mean age at diagnosis was 4,1 ± 2,6 years (1 year-13 years). Three patiets (37,5%) were presented with vomitting (Table 1). Three (37,5%) had nonspecific upper respiratory tract symptoms at admission and recurrent lung infection history. In two (25%) the hernia was discovered incidentally. Diagnosis was reached by two sided plain chest X-Ray and/or computed tomography (Fig. 2). Down syndrome was noted in 3 (38%) patients.

**Table 1:** Demographics and clinical presentation. CXR - plain chest X-Ray, CT-computed tomography.

Case	Gender	Age at diagnosis (year)	Clinical presentation	Diagnosis	Chromosomopathy
**1**	Male	1	Vomiting	CXR	Down sydrome
**2**	Male	1	Incidentally	CXR	No
**3**	Male	2	Incidentally	CXR,CT	No
**4**	Male	2	Vomiting	CXR,CT	Down sydrome
**5**	Male	3	Frequent lung infection	CXR,CT	No
**6**	Male	4	Frequent lung infection	CXR,CT	No
**7**	Male	7	Vomiting	CXR	No
**8**	Male	13	Frequent lung infection	CXR,CT	Down sydrome

In all cases, the correction was performed using transabdominal laparoscopic-assisted technique. At the operation, defect and sac of hernia was checked. Seven (87,5%) patients had left-sided and one (12,5%) patient had bilateral hernia (Table 2). Hernia sacs were present in all of the patients. None of the sacs were removed. The hernial contents were only omentum in two patients, omentum and colon in six and colon, omentum and stomach in one patient. There were no intraoperative complications. The average discharge time was 2.6 days. There is only one suture reaction in subcutaneous tissue at postoperative 2 months. There were no complications or recurrence in the mean 19,2 ± 15,8 months (6–42 months) follow-up period.

**Table 2:** Perioperative and follow-up details. CXR-plain chest X-Ray.

Case	Age at surgery (year)	Laterality	Hernia contents	Suture	Length of hospital stay (days)	Residual cavity (CXR)	Recurrence	Follow-up (months)
**1**	1	Left	Stomach, colon, omentum	Nonabsorbable	4	No	No	42
**2**	1	Left	Omentum	Nonabsorbable	2	–	No	26
**3**	2	Left	Colon, omentum	Nonabsorbable	2	No	No	15
**4**	2	Left	Stomach, colon, omentum	Nonabsorbable	3	–	No	32
**5**	3	Left	Omentum	Nonabsorbable	2	No	No	
**6**	4	Left	Colon, omentum	Nonabsorbable	2	No	No	13
**7**	7	Bilateral	Colon, omentum	Nonabsorbable	2	No	No	22
**8**	13	Left	Colon, omentum	Nonabsorbable	4	No	No	6

## 4 Discussion

Congenital MHs are a rare form of diaphragmatic hernia that make up 2–4% of all congenital diaphragmatic hernias [3,4]. MHs have a variety of clinical presentations ranging from severely life-threatening at the time of birth to remaining asymptomatic until adulthood. It can be discovered either incidentally or as a result of vague gastrointestinal complaints; more commonly, it causes respiratory symptoms, which can be severe during infancy [8,13]. This was the case in our series as the majority of our patients presented with repeated attacks of chest infections and with vomiting and abdominal pain in acute situation.

Chromosomal disorders and congenital abnormalities with MH have been reported to be around 20% in the literature [7,14]. As in earlier reports, Down syndrome was a frequent association (38%) [15,16]. In present study, three of our patients (37,5%) had Down syndrome. Two of the 3 patients with Down syndrome had vomiting and the other had a history of frequent lung infection. MH should be considered as the differential diagnosis in patients with Down sydrome that admitted with frequent recurrent lung infections or vomiting.

It is generally accepted that surgical repair of Morgagni hernia should be performed even in asymptomatic children to prevent major complications like intestinal obstruction, volvulus or perforation. For many years, thoracotomy and specially laparotomy have been the standard surgical approaches. After the first laparoscopic repair of MH by Kuster et al. [17] in 1992, minimally invasive techniques became rapidly accepted as elected approaches in the repair of MH [[18], [19], [20]].

Thoracoscopic approach of thoracic surgeons has not received any interest in pediatric surgery because of the necessity of opening hernia sac, narrowed study area, ineffectiveness in bilateral cases and the risk of peroperative complications [21]. As transabdominal approach, a variety of techniques to repair Morgagni hernia laparoscopically have been described in the literature using either primary closure with a continuous suture, interrupted suture, or using a mesh [22,23]. Our patients had variable sizes of hernia defects but in none of them there was a need to use a patch because the defect could be closed without tension.

The laparoscopic interrupted or continuous suture technique to repair Morgagni hernia is complicated and time consuming. In this study, in all cases, the correction was performed using transabdominal laparoscopic-assisted technique using three ports and when sutures were performed with, separated, percutaneous, ‘‘U’’ shaped, nonabsorbable stitches, through the full thickness of the anterior abdominal wall and the knots were tied in the subcutaneous tissue by a single incision. Cosmetic appearance was obtained by removing all sutures from the same skin incision. Cosmetic appearance could be further improved by making it from single-port. The full thickness stitches allows for maximum strength repair. By contrast, anchoring the sutures in the back of the sternum and costal margin is technically challenging and the fascia may not be strong enough. There were no recurrence in this series. We believe that full thickness stitches is useful for preventing recurrence.

Another controversial issue at the time of repair is whether to excise or leave the associated hernia sac [6,8,24,25]. Excision is suggested in order to reduce recurrence rate, but it may be potentially dangerous such as possible injury of the pericardium, pleura, or phrenic nerve that might be associated with the hernia sac excision. In present study, hernia sac was not removed in any patient and we had no adverse events leaving the hernia sac in place and no effect on recurrence. And also there were no residual cavity in chest X-ray in follow up period.

In conclusion, laparoscopic-assisted repair of MH using sutures including the full thickness of anterior abdominal wall and extracorporeal knots proved to be effective, safe, and reliable in children. It should be the first choice because of fast recovery and better cosmetic results. In this series, it was seen that leaving the hernia sac had no effect on early and late complications. Leaving the hernia sac may prevent potential complications due to unnecessary dissection.

## Informed consent

Informed consents were obtained from the patients.

## Provenance and peer review

Not commissioned, externally peer reviewed.

## Ethical approval

Authors declared that the research was conducted according to the principles of the World Medical Association Declaration of Helsinki “Ethical Principles for Medical Research Involving Human Subjects”, (amended in October 2013).

## Sources of funding

The authors declared that this study has received no financial support.

## Author contribution

Author Contributions: Concept – A.İ.A., G.G.; Design - G.G.; Supervision- A.İ.A.; Resources - A.İ.A., G.G., O.H.K; Materials – A.İ.A., G.G.,O.H.K; Data Collection and/or Processing – A.İ.A., G.G., O.H.K; Analysis and/or Interpretation - G.G.; Literature Search – A.İ.A, G.G.; Writing Manuscript – A.İ.A., G.G.; Critical Review - G.G.

## Registration of research studies

1. Name of the registry: Researchregistry
2. Unique Identifying number or registration ID: 5421
3. Hyperlink to the registration (must be publicly accessible): https://www.researchregistry.com/browse-the-registry#home/registrationdetails/5e6a931f29050500186e3538/

## Guarantor

Dr. Ali İhsan Anadolulu.

Dr.Gonca Gerçel.

## Declaration of competing interest

The authors have no conflict of interest to declare.
